# Ophthalmologic problems correlates with cognitive impairment in patients with Parkinson's disease

**DOI:** 10.3389/fnins.2022.928980

**Published:** 2022-10-06

**Authors:** Chao Zhang, Qian-qian Wu, Ying Hou, Qi Wang, Guang-jian Zhang, Wen-bo Zhao, Xu Wang, Hong Wang, Wei-guo Li

**Affiliations:** ^1^Department of Neurosurgery, Qilu Hospital of Shandong University, Jinan, China; ^2^Institute of Brain and Brain-Inspired Science, Shandong University, Jinan, China; ^3^Department of Neurology, Qilu Hospital of Shandong University, Jinan, China; ^4^Department of Gerontology, Shandong Provincial Qianfoshan Hospital, Jinan, China; ^5^Department of Neurology, Weifang People's Hospital, Weifang, China; ^6^Department of Ophthalmology, Qilu Hospital of Shandong University, Jinan, China

**Keywords:** Parkinson's disease, visual dysfunction, cognitive impairment, visual impairment in Parkinson's disease questionnaire, deep brain stimulation

## Abstract

**Objective:**

Visual impairment is a common non-motor symptom (NMS) in patients with Parkinson's disease (PD) and its implications for cognitive impairment remain controversial. We wished to survey the prevalence of visual impairment in Chinese Parkinson's patients based on the Visual Impairment in Parkinson's Disease Questionnaire (VIPD-Q), identify the pathogens that lead to visual impairment, and develop a predictive model for cognitive impairment risk in Parkinson's based on ophthalmic parameters.

**Methods:**

A total of 205 patients with Parkinson's disease and 200 age-matched controls completed the VIPD-Q and underwent neuro-ophthalmologic examinations, including ocular fundus photography and optical coherence tomography. We conducted nomogram analysis and the predictive model was summarized using the multivariate logistic and LASSO regression and verified *via* bootstrap validation.

**Results:**

One or more ophthalmologic symptoms were present in 57% of patients with Parkinson's disease, compared with 14% of the controls (χ^2^-test; *p* < 0.001). The visual impairment questionnaire showed good sensitivity and specificity (area under the curve [AUC] = 0.918, *p* < 0.001) and a strong correlation with MoCA scores (Pearson *r* = −0.4652, *p* < 0.001). Comparing visual impairment scores between pre- and post-deep brain stimulation groups showed that DBS improved visual function (*U*-test, *p* < 0.001). The thickness of the retinal nerve fiber layer and vessel percentage area predicted cognitive impairment in PD.

**Interpretation:**

The study findings provide novel mechanistic insights into visual impairment and cognitive decline in Parkinson's disease. The results inform an effective tool for predicting cognitive deterioration in Parkinson's based on ophthalmic parameters.

## Introduction

Visual impairment is a common non-motor symptom (NMS) in patients with Parkinson's disease (PD), affecting up to 78% of patients with PD (Hamedani et al., 2020). Although vision is an important determinant of quality of life, visual impairment is often underreported by patients and overlooked by their physicians (Ekker et al., 2017). Deficits in contrast sensitivity, color discrimination, and stereopsis are widespread in patients with PD and are associated with the risk of cognitive decline (Leyland et al., 2020; Murueta-Goyena et al., 2021). These parameters are not routinely assessed by ophthalmologists and neurologists. Visual dysfunction in PD is subtle and unlikely to be captured outside of the research setting. We introduced the Visual Impairment in Parkinson's Disease Questionnaire (VIPD-Q) (Borm et al., 2020) to provide insight into the relationship between ophthalmologic problems and cognitive impairment in patients with PD.

The role of visual dysfunction as a biomarker of disease and a predictor of cognitive impairment in PD has been reported in previous studies (Weil et al., 2016). These studies usually ascribe visual deficits to intracranial impairment. As the understanding of the gut–brain axis deepens, the knowledge basis for visual dysfunction in PD will likely change over time. We conducted metatranscriptomic sequencing and 16s rDNA sequencing in PD patients with and without visual impairment to identify the relationship between ophthalmology dysfunction and microbiota dysbiosis in patients with PD. To our knowledge, we have conducted the first study focusing on this issue.

We utilized optical coherence tomography (OCT) and machine learning software to analyze the ocular characteristics of patients with PD, such as retinal nerve fiber layer (RNFL) thickness and microvascular density in the fundus. These indices have been demonstrated to correlate with cognitive status in PD within a few pioneering studies (with relatively small sample sizes ranging from *n* = 17 to *n* = 63); only a limited number of ophthalmologic symptoms have been evaluated in PD (Kwapong et al., 2018; Murueta-Goyena et al., 2019, 2021).

We aimed to systematically determine the application value of VIPD-Q in patients with PD and explore the link between ophthalmology dysfunction and cognitive impairment.

## Methods

### Study design

We used the VIPD-Q screening questionnaire to assess ophthalmologic symptoms and UPDRSIII and MoCA scores in patients with PD (Borm et al., 2020). The questionnaire was administered by two university hospitals (Qilu Hospital, First Affiliated Hospital) and scored by two clinicians (one ophthalmologist, HW and one neurologist, CZ) between June 2019 and July 2021. Participants underwent neuro-ophthalmologic examinations, including ocular fundus photography, automated perimetry, and OCT. Patients undergoing deep brain stimulation (DBS) took an extra VIPD-Q 3 months after the operation. Exclusion criteria included secondary causes of parkinsonism, prior brain surgery (except DBS), glaucoma, intraocular surgery, diabetes, and other diseases that affected the visual field or neurologic systems, and the current use of medications that affected visual function.

### Ethical considerations

The study was conducted in accordance with the principles of the Declaration of Helsinki. The Ethics Committees at the Qilu Hospital (protocol KYLL-202008-065) and the First Affiliated Hospital of Shandong First Medical University (protocol S569) approved this study. Written informed consent was obtained from all participants.

### Measures

RNFL and retinal thickness were measured using the Spectralis OCT device (CIRRUS 5000, Carl Zeiss, Oberkochen, Germany). Scans were performed by the same operator (HW). Image acquisition was conducted using the TruTrack eye-tracking technology that recognizes, locks onto, and follows the patient's retina. Ocular fundus photography was performed by an ophthalmologist using a digital ocular fundus camera (VISUCAM 224, Carl Zeiss, Germany) in both eyes in patients with PD. At least one reliable picture per eye was acquired for each patient. Images of ocular fundus vessels were extracted based on the U-Net model (github.com/orobix/retina-unet#retina-blood-vessel-segmentation-with-a-convolution-neural-network-u-net) (Liskowski and Krawiec, 2016) using a novel software (github.com/jellygrey/OVE/tree/master/bin). Vessel pictures were measured *via* the Angiotool software (version 0.6a) to obtain vessel-related data (Segarra et al., 2018).

We performed perivascular spaces (PVS) quantification in slices containing the maximum amount of PVS in the basal ganglia (BG) region using ITK-SNAP software (version 3.8; the University of Pennsylvania, the University of North Carolina at Chapel Hill) (Shen et al., 2021). Boundaries of all identified PVS were delineated manually. This software automatically provides the voxel number for identified PVS in each region. PVS volume was calculated as the sum of individual volumes of the identified PVS in each region per mm^3^. We then obtained PVS counts and volumes for each region.

### Nomogram construction and validation

Multivariable logistic regression was applied according to sex, age, disease duration, VIPD-Q score, RNFL thickness, average vessel percentage, RBD status, hyposmia, and constipation. The “glmnet” package was used to conduct LASSO regression to screen meaningful variants, which were verified by bootstrap validation. A nomogram was generated *via* the “regplot” R package as a quantitative tool for predicting the risk of cognitive impairment. Consistency between model predictions and clinical outcomes was assessed using the concordance index (C-index). A calibration plot was generated to evaluate the accuracy of MoCA predictions. Decision curve analysis (DCA) was applied to evaluate nomogram performance using the “rmda” R package.

### Statistical analysis

Statistical analyses were performed using the Prism software (version 8.0.1; San Diego, CA, USA), R (version 3.6.1), and RStudio (version 1.2.1335). Patients with PD were compared with controls using χ^2^-tests for categorical variables (education, sex, comorbidity, and visual impairment) and Mann-Whitney *U*-tests for nonparametric continuous variables (age, VIPD-Q score, score per domain, MoCA score, UPDRSIII score, and levodopa equivalent dose [LED]). To explore correlations, we fit linear models and calculated Spearman's *r*-value. Univariate logistic regression was used to evaluate the statistical significance (defined as *p* < 0.05) of associations between visual function parameters and motor and non-motor symptoms.

## Results

### Participant characteristics

A total of 405 participants completed the questionnaire: 205 patients with PD and 200 age-matched controls (1:1 ratio). Ophthalmologic symptoms were present in 57% of patients with PD (vs. 14% of the controls; χ^2^-test: *p* < 0.001). Baseline characteristics and prevalence of ophthalmologic symptoms are summarized in Table 1. The groups were well balanced for age and comorbidity.

**Table 1:** Participant characteristics and Prevalence of ophthalmologic symptoms.

	**PD (*n* = 205)**	**Con (*n* = 200)**	***P*** **-value**
Men, n (%)	108, (53)	106, (53)	NS
Age, y, median (IQR) [range]	63 (12) [21–82]	64 (10) [20–74]	NS
Disease duration, y, median (IQR) [range]	8 (5) [2–30]	NA	NA
Levodopa dose equivalent, mg, median (IQR) [range]	562 (375) [0–2,000]	NA	NA
Hypertension	45, (22)	36, (18)	NS
Stroke	16, (8)	14, (7)	NS
COPD	44, (22)	44, (22)	NS
Uses visual aid, n (%)	72, (35)	24, (12)	<0.001
Using a walking aid outside, n (%)	70, (34)	14, (7)	<0.001
Falls present (last 6 months)	20, (10)	4, (2)	<0.001
**Ophthalmologic symptoms reported weekly or daily**, ***n*** **(%)**			
**Ocular surface**			
1. I have blurry vision when I read or work on a computer.	93, (45)	68, (34)	<0.001
2. I have a burning sensation or gritty feeling in my eyes	24, (12)	16, (8)	<0.001
3. I have mucus/slime or particles in my eyes or eyelids.	22, (11)	16, (8)	<0.001
4. I have watery eyes.	31, (15)	34, (17)	<0.001
**Intraocular**			
1. When I read, some letters disappear	24, (12)	16, (8)	<0.001
2. Lines that should be straight appear to be wavy or blurred.	26, (13)	6, (3)	<0.001
3. I won't go out alone in the evening or at night because my night vision is insufficient.	21, (10)	10, (5)	<0.001
4. When I drive at night, the oncoming headlights cause more glare than before.	64, (31)	38, (19)	<0.001
**Oculomotor**			
1. Quick movements are hard to follow with my eyes.	112, (55)	32, (16)	<0.001
2. I have double vision.	118, (58)	40, (20)	<0.001
3. I can read better with one eye closed.	16, (8)	20, (10)	<0.001
4. I have trouble with depth perception. I find it hard to say which one of 2 objects is closer.	18, (9)	8, (4)	<0.001
**Optic nerve**			
1. Colors seem to be paler than before.	18, (9)	16, (8)	<0.001
2. I can't read plain text on a colored or gray background.	54, (26)	22, (11)	<0.001
3. I run into objects or people or feel that parts of my visual field are missing.	14, (7)	8, (4)	<0.001
4. I have problems with rapid changes of light intensity.	60, (29)	10, (5)	<0.001
5. I see things that other people do not see (hallucinations).	60, (29)	12, (6)	<0.001

### VIPD-Q correlated with UPDRSIII, and MoCA scores

**Figure 1:**
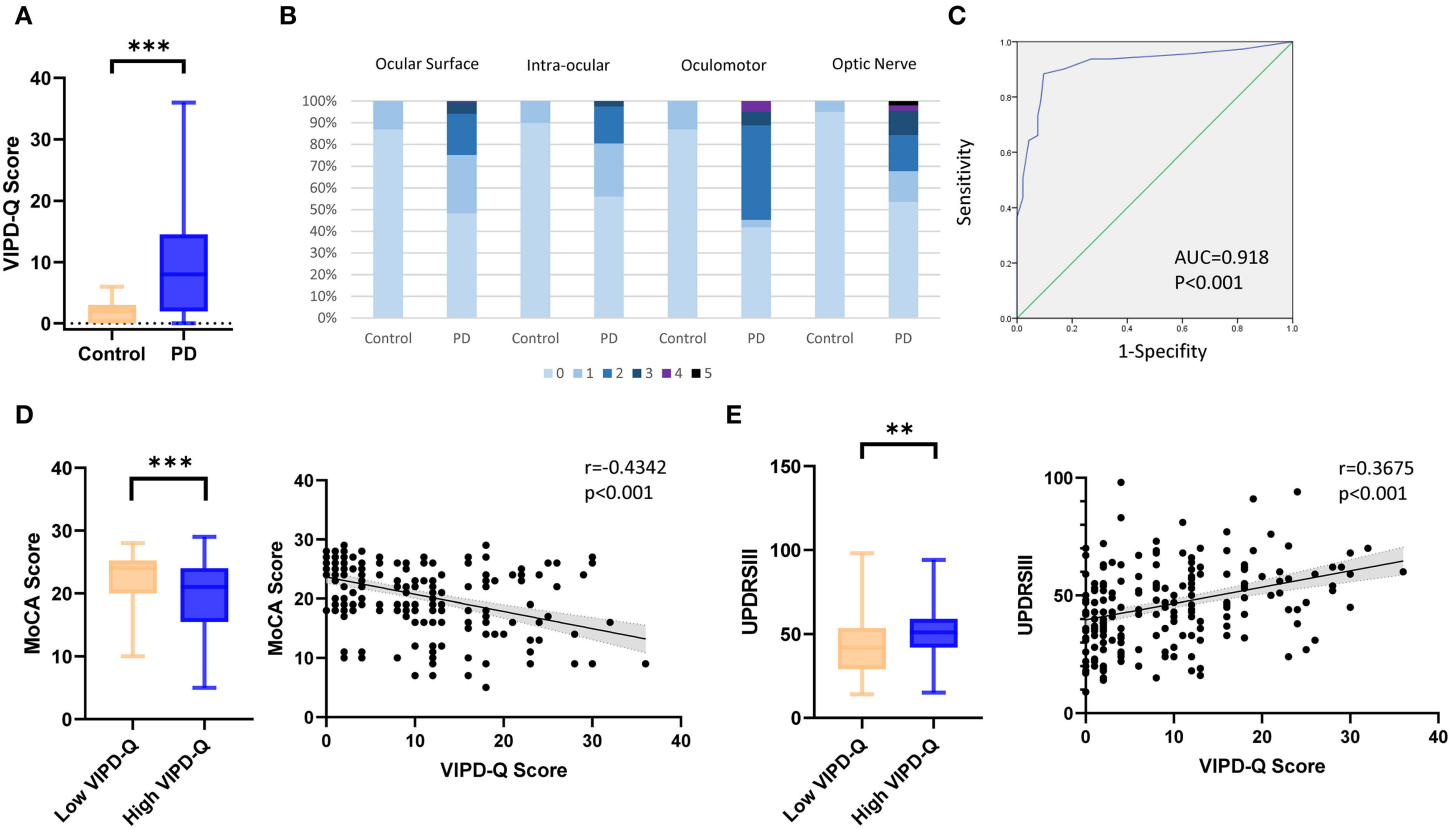
VIPD-Q scores for patients with PD in relation to MoCA and UPDRSIII scores. **(A)** Boxplot of the median total VIPD-Q score in the PD and control groups. **(B)** Number of ophthalmologic symptoms reported per domain (compared between patients with PD and controls). **(C)** The validation of sensitivity and specificity for the VIPD-Q *via* an ROC curve. **(D,E)** The VIPD-Q score was correlated with MoCA and UPDRSIII scores in PD.**p < 0.01 and ***p < 0.001. PD, Parkinson's disease; ROC, receiver operating characteristic; VIPD-Q, Visual Impairment in Parkinson's Disease Questionnaire; UPDRS III, Unified Parkinson's Disease Rating Scale Section III; MoCA, Montreal Cognitive Assessment.

The PD group experienced more ophthalmologic symptoms, reflected by median total VIPD-Q scores (Mann-Whitney *U*-test, *p* < 0.001; Figure 1A). Figure 1B shows the number of ophthalmologic symptoms per domain (PD vs. control, χ^2^-test: ocular surface, 51.7 vs. 13%; intraocular domain, 43.9 vs. 10%; oculomotor domain, 58.1 vs. 13%; optic nerve domain, 46.3 vs. 5%; *p* < 0.001).

Based on a previous study (Borm et al., 2019), we reran tests to identify the sensitivity and specificity of the VIPD-Q in the Chinese population. A receiver operating characteristic (ROC) curve indicated good sensitivity and specificity (AUC = 0.918, *p* < 0.001; Figure 1C); this result was verified using the Hosmer–Lemeshow test (*p* = 0.247). According to Youden's index, the optimal ROC cut-off value was 7 (sensitivity, 88.4%; specificity, 90.3%; Youden's index, 0.787). We divided the population into a low VIPD-Q group (score < 7; PD with normal visual function) and a high VIPD-Q group (score ≥ 7; PD with impaired visual function). The high VIPD-Q group showed higher UPDRSIII scores (Mann-Whitney *U*-test, *p* = 0.0013) and lower MoCA scores (Mann-Whitney *U*-test, *p* = 0.0004) than the low VIPD-Q group (Figure 1D). VIPD-Q scores were positively associated with UPDRSIII scores (Pearson *r* = 0.3675, *p* < 0.0001) and negatively associated with MoCA scores (Pearson *r* = −0.4342, *p* < 0.0001; Figure 1E).

### STN-DBS and non-motor symptoms affected VIPD-Q scores

**Figure 2:**
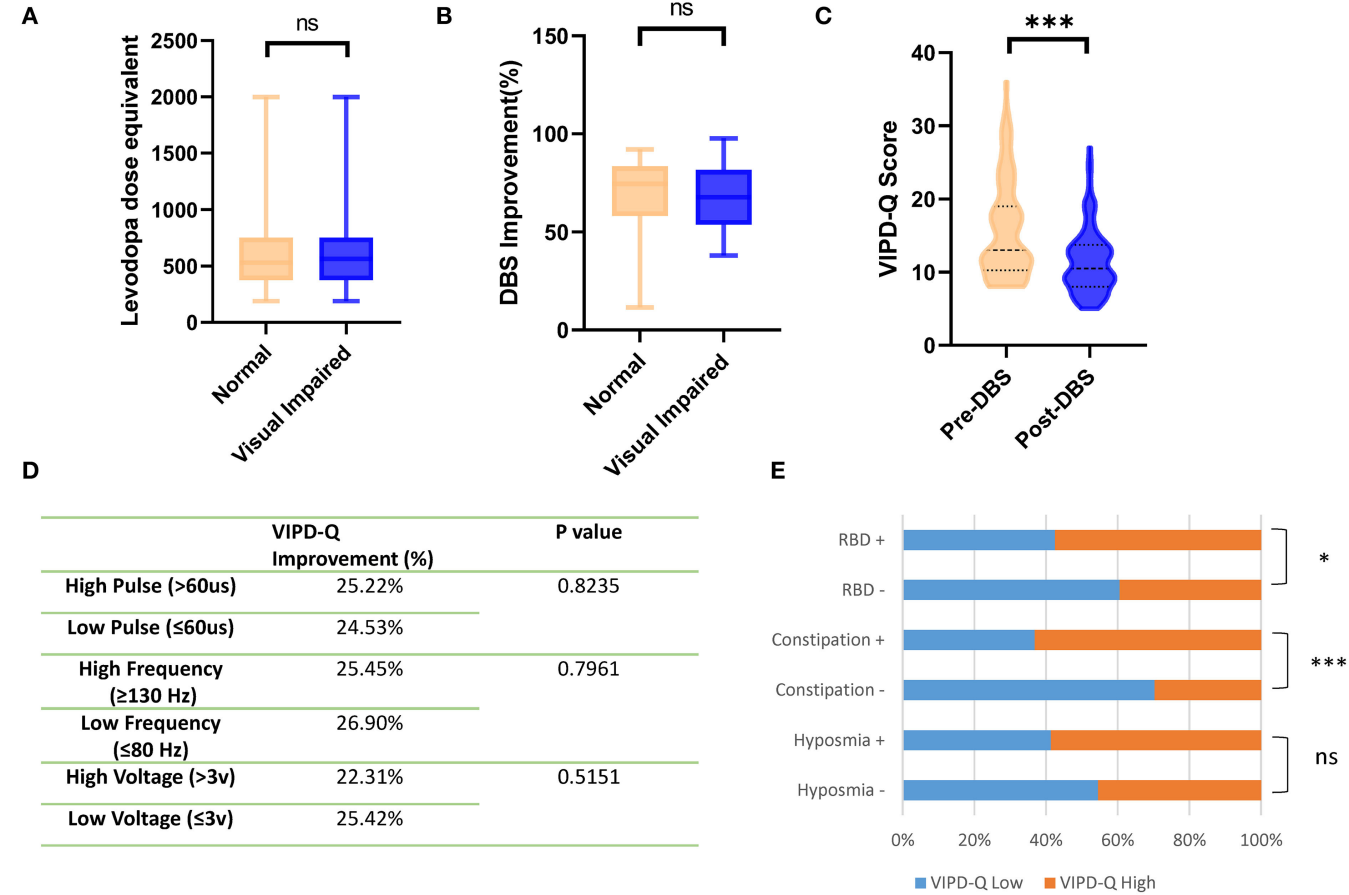
DBS and non-motor symptoms affected VIPD-Q scores in PD. **(A,B)** Groups with different VIPD-Q score presented similar LED and DBS improvements **(C)**. DBS improved VIPD-Q scores. **(D)** Stimulation parameter changing did not affect the VIPD-Q score. **(E)** PD patients with RBD or constipation had higher percentage of visual impairment (VIPD-Q>6). ns, no significant and ***p < 0.001. DBS, deep brain stimulation; RBD rapid eye movement (REM) sleep behavior disorder; VIPD-Q, Visual Impairment in Parkinson's Disease Questionnaire.

To determine factors influencing VIPD-Q scores in PD, we compared the LED between PD patients with and without visual impairment. No statistically significant differences were found (*t*-test, *p* = 0.2689; Figure 2A). As DBS is an option for PD treatment, we compared the improvement efficiency between the high- and low-VIPD-Q groups. The groups had similar DBS improvement (*t*-test, *p* = 0.7503; Figure 2B). However, at their 3-month follow-up, the post-DBS group showed a clear decrease in the VIPD-Q score as compared to the pre-DBS group (13.0 vs. 10.50, Mann-Whitney *U*-test, *p* < 0.001; Figure 2C). As low-frequency stimulation was reported to improve axial symptoms more effectively than high-frequency stimulation (Xie et al., 2017), we compared groups with different stimulation parameters *via t*-tests; no differences were found (Figure 2D).

Other NMS were shown to affect VIDP-Q scores in our study. The RBD-positive group had a higher visual impairment percentage (score > 6; chi-square: *p* = 0.0139), especially in the constipation-positive group (*p* < 0.0001; Figure 2E). Hyposmia did not seem to affect visual function (chi-square, *p* = 0.0599), although the nerve fibers were anatomically close to each other.

### Thinner RNFL was found in PD patients with visual impairment and cognitive dysfunction

**Figure 3:**
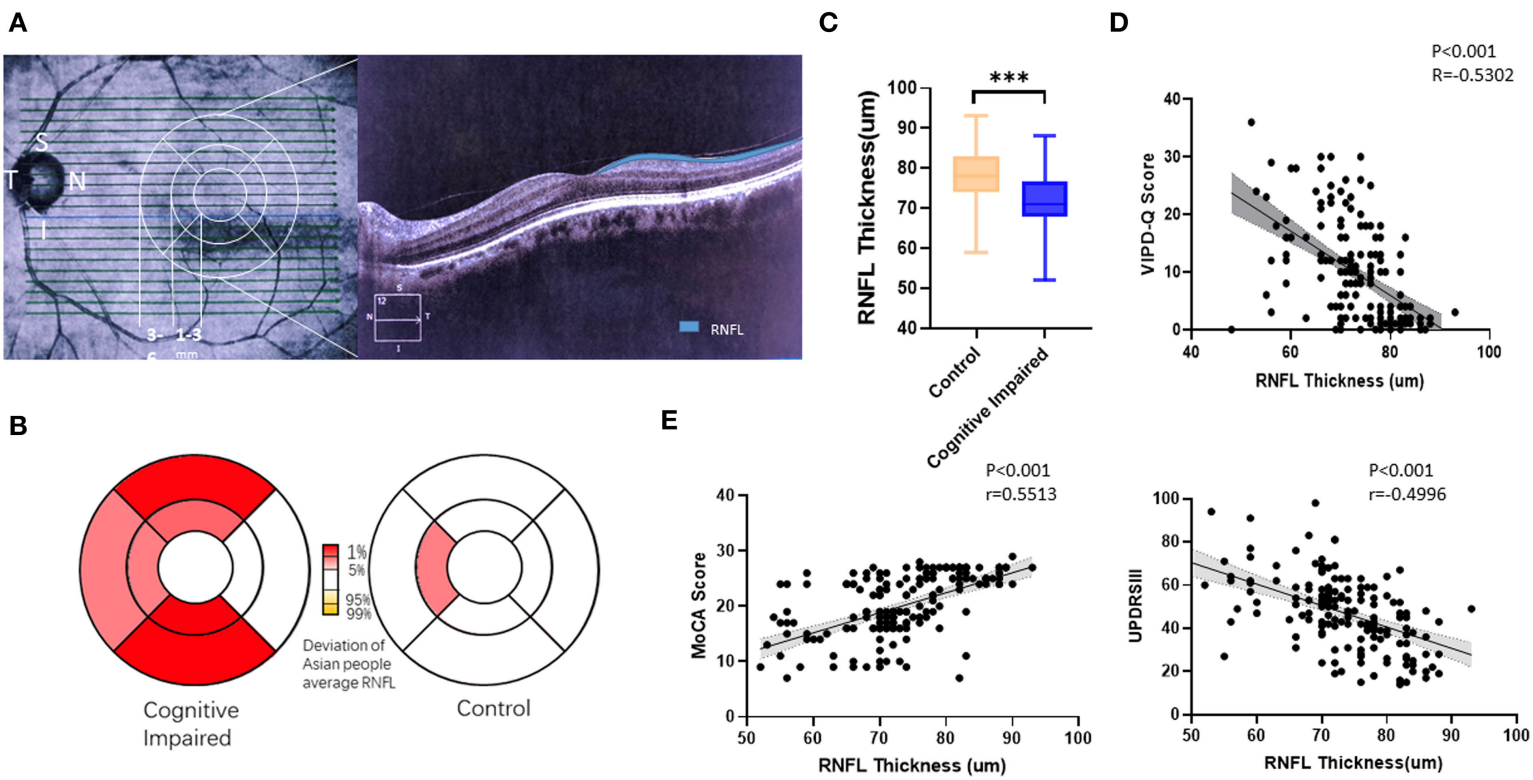
Thinner RFNL in the ocular fundus was found in the cognitively impaired group. **(A)** Representative picture of a measured RNFL. **(B)** Deviation map of RNFL indicating thinner thickness. **(C)** PD patients with cognitive impairment had, on average, a thinner RNFL. **(D)** VIPD-Q was related to RNFL thickness. **(E)** RNFL was related to MoCA and UPDRSIII scores. **p < 0.01, ***p < 0.001, and *r* = Pearson *r*. RNFL, retinal nerve fiber layer; PD, Parkinson's disease; VIPD-Q, Visual Impairment in Parkinson's Disease Questionnaire; UPDRS III, Unified Parkinson's Disease Rating Scale Section III; MoCA, Montreal Cognitive Assessment.

Patients with PD were divided into a cognitively impaired group (MoCA <26) and a normal control group (MoCA≥26). OCT examination was conducted to determine the thickness of the RNFL (Figure 3A). The deviation map for RNFL is summarized in Figure 3B. Results indicate that the superior and inferior directions in the perifovea region presented a higher deviation in the visually impaired group compared to the Asian average RNFL thickness (Figure 3B). Thinner RNFL in the ocular fundus was found in the cognitively impaired group (*t*-test, *p* < 0.001; Figure 3C). We examined VIPD-Q scores according to RNFL thickness *via* Spearman correlation analysis (*p* < 0.001, Pearson *r* = −0.5302; Figure 3D). Correlations between RNFL, MoCA, and UPDRSIII were analyzed, with positive associations between RNFL thickness and MoCA score (*p* < 0.001, Pearson *r* = 0.5513; Figure 3D), and a negative correlation was found between UPDRSIII score and RNFL thickness (*p* < 0.001, Pearson *r* = −0.4996; Figure 3E).

### Vessel density in ocular fundus correlated with MoCA score and PVS number in PD

**Figure 4:**
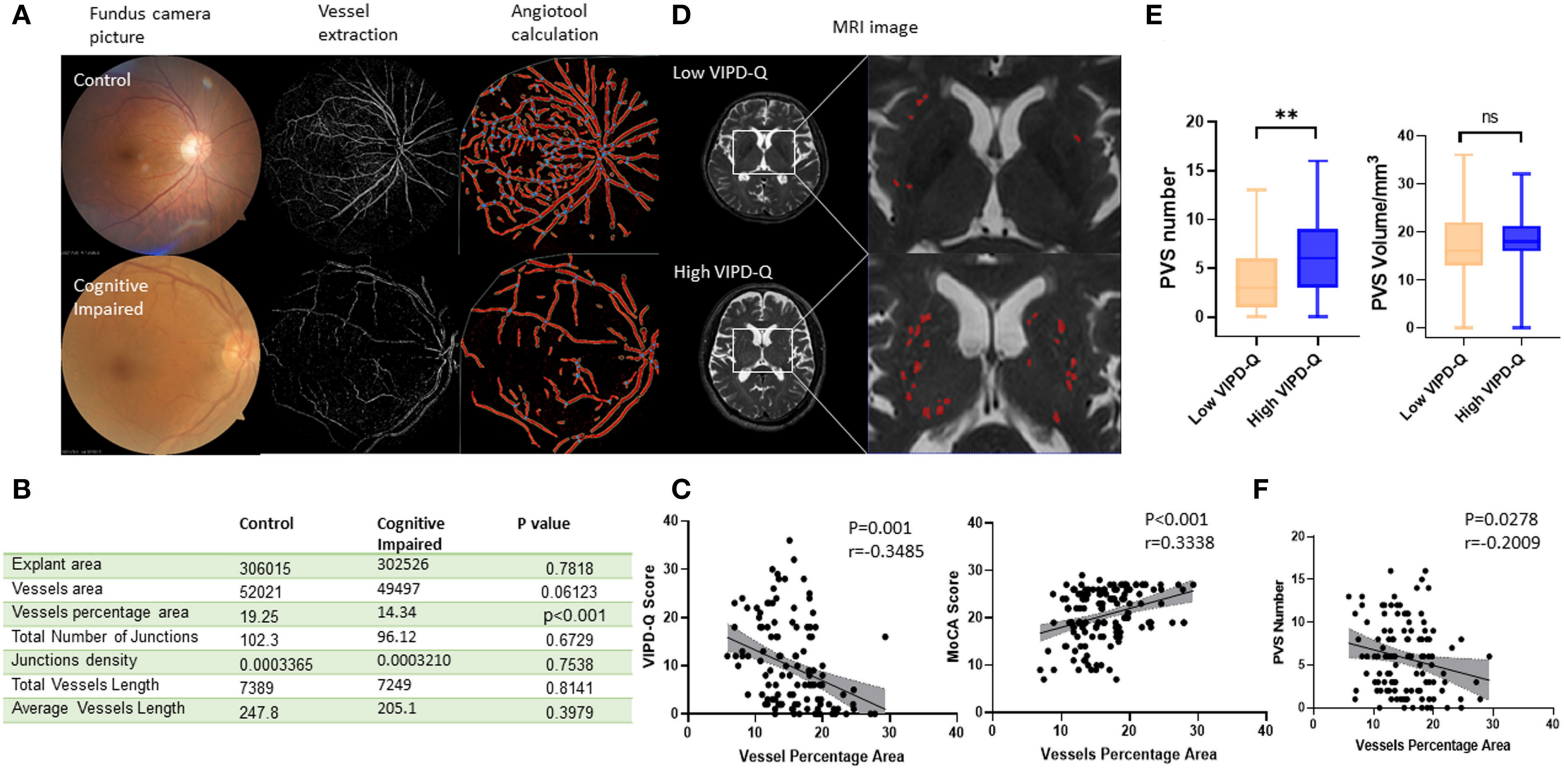
Vascular density in ocular density predicted PVS number and cognitive status in PD. **(A)** Typical procedure for calculating vascular density in groups with or without cognitive impairment. **(B)** A table of vascular-related parameters in ocular fundus analyzed by angiotool. **(C)** The vessel percentage area was correlated with VIPD-Q and MoCA scores. **(D)** Representative MRI images for VIPD-Q high and low groups. **(E)** PVS count showed a discrepancy between VIPD-Q groups. **(F)** The vessel percentage area was correlated with PVS counts. **p < 0.01, ***p < 0.001, and *r* = Pearson r. MRI, magnetic resonance imaging; PD, Parkinson's disease; PVS, perivascular spaces; VIPD-Q, Visual Impairment in Parkinson's Disease Questionnaire; MoCA, Montreal Cognitive Assessment.

The vessels in the ocular fundus were extracted and analyzed *via* software (Figure 4A). Data showed the vessel percentage area (VPA), which indicated that vessel density in the ocular fundus was obviously lower in the cognitively impaired group (*t*-test, *p* < 0.001; Figure 4B). We then evaluated the relation between VIPD-Q scores and VPA *via* Spearman correlation analysis (*p* = 0.001, Pearson *r* = −0.3485; Figure 4C). Correlations between VPA and MoCA were analyzed, with positive associations between VPA and MoCA scores (*p* < 0.001, Pearson *r* = 0.3338; Figure 4C). When retrospect the MRI images, PVS counts were likewise abnormal in the visually impaired group (Figure 4D). PVS counts were higher in the high VIPD-Q group than in the control group (*t*-test, *p* = 0.006; Figure 4E); PVS volume did not differ (*t*-test, *p* = 0.2679; Figure 4E). We found negative correlations between VPA and PVS count (*p* = 0.0278, Pearson *r* = −0.2009; Figure 4F). Thus, eye examination may open a window for observing the process of intracranial disease noninvasively.

### Nomogram based on ophthalmic parameters for predicting cognitive impairment risk

**Figure 5:**
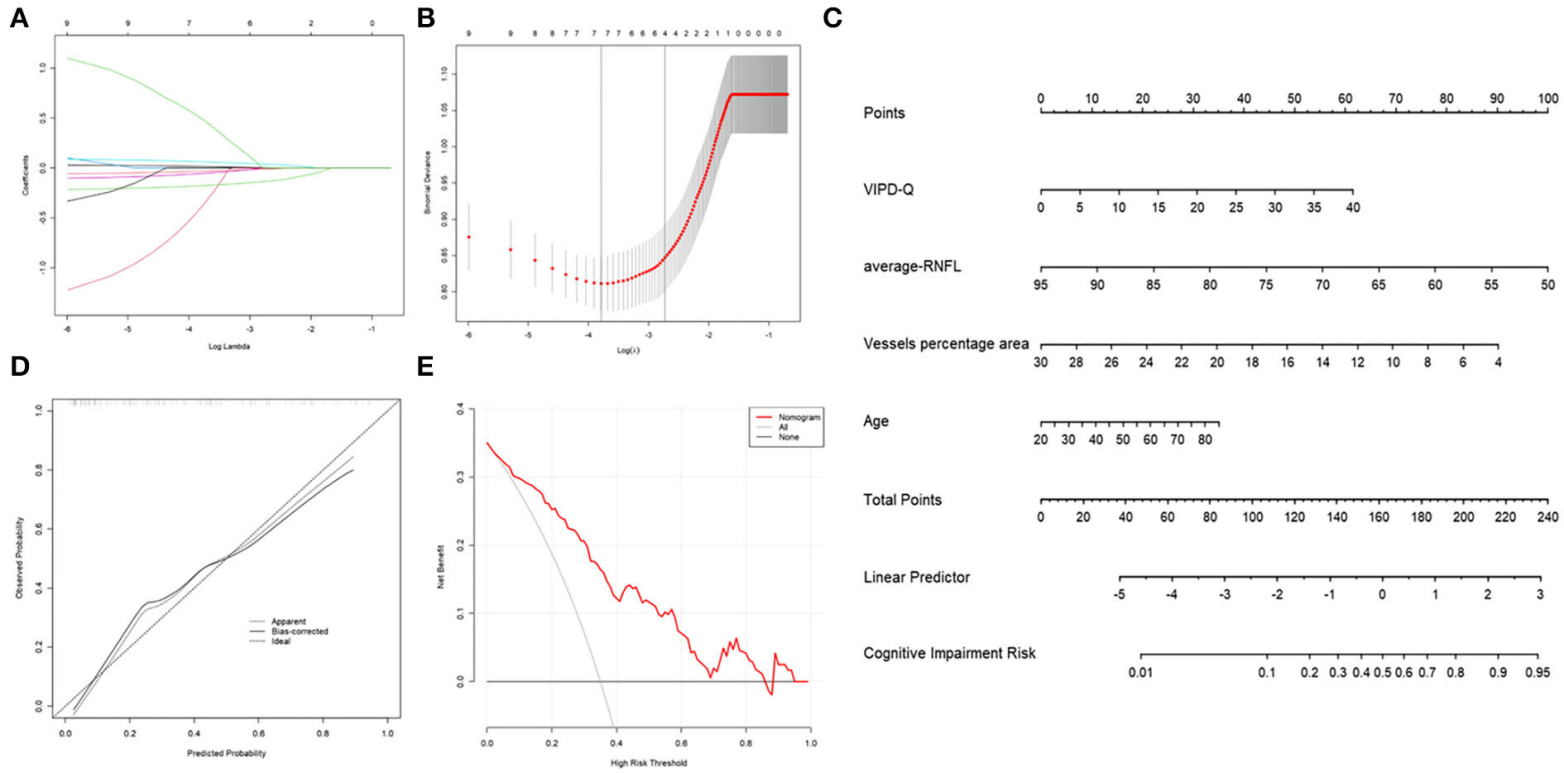
Nomogram of ophthalmic parameters predicting the risk of cognitive impairment in PD. **(A)** LASSO regression analysis was conducted to screen out MoCA score-related parameters. **(B)** Bootstrap validation was performed to verify the screened event. **(C)** A nomogram predicting the risk of cognitive impairment in PD was constructed based on ophthalmic parameters. **(D)** A calibration curve was drawn to decrease the bias of the nomogram. **(E)** A DCA curve showed that the nomogram would benefit patients with PD by accurately predicting the risk of cognitive impairment. DCA, decision curve analysis; PD, Parkinson's disease; MoCA, Montreal Cognitive Assessment.

To evaluate the prognostic effect of ophthalmologic factors on cognitive impairment, numerous events (age, disease duration, sex, VIPD-Q score, RNFL thickness, vessel percentage area, etc.) were analyzed using multivariate logistic and LASSO regression (Figure 5A). Age, VIPD-Q score, RNFL thickness, and VPA were screened and verified *via* a bootstrap validation (Figure 5B). A nomogram was constructed based on these events (Figure 5C). The point scale in the nomogram was utilized to generate points for these variables; the risk of cognitive impairment (MoCA score < 26) was determined by accumulating the total points for all variables. The C-index of this nomogram was good, reaching 0.8373 (95% CI: 0.766–0.908). Consistency between predicted and observed clinical outcomes for patients with PD was confirmed *via* the calibration plot (Figure 5D). DCA showed a higher overall net benefit by applying the nomogram than either the “treat all” or the “treat none” approach within a range of threshold probabilities >10% (Figure 5E). These findings demonstrate that the ophthalmic event-based nomogram is an optimal model for predicting cognitive impairment in patients with PD in clinical management.

## Discussion

In this study, we demonstrated that the VIPD-Q could be applied to the Chinese population. Results revealed that patients with PD have a higher prevalence of ophthalmologic symptoms, as reflected by a high VIPD-Q score, suggesting that PD itself or its treatment has an effect on an ophthalmologic function beyond the normal aging process. These results are in agreement with a previous European study (Borm et al., 2020).

To verify the sensitivity and specificity of the VIPD-Q, we plotted the ROC curve and calculated the cut-off value. We were thus able to screen PD patients with visual impairment in a large population. To our knowledge, the ideal cut-off value has not been reported previously and may provide a reference for similar studies.

An earlier study reported that visual impairment is a sensitive marker for PD (Weil et al., 2016); one study showed better discriminatory power for the early diagnosis of PD than any other NMS (Diederich et al., 2010). Our results demonstrated the predictive value of VIPD-Q for MoCA and UPDRSIII scores, echoing earlier findings that visuospatial motion perception and RNFL thickness correlate with movement disorders in PD (Beylergil et al., 2021; Murueta-Goyena et al., 2021).

The VIPD-Q scores indicated that PD or its treatment influence visual function. Studies report that oral levodopa rescues retinal morphology and improves visual function in amblyopia (Lee et al., 2019; Wang et al., 2020). We assumed that PD patients without ophthalmologic symptoms would benefit from a higher levodopa dose. However, our results conflict with this hypothesis, as different VIPD-Q score groups shared similar levodopa equivalents. DBS is another widespread treatment for PD (Weil et al., 2016), and an attractive yet less explored area is whether DBS could help patients with PD avoid visual deficits. Studies report that DBS improves saccades, smooth eye movements, and visual scanning (Murueta-Goyena et al., 2019). Others showed contradictory results (a little effect was observed after stimulation) (Kwapong et al., 2018). We evaluated the effects of DBS on ophthalmologic symptoms using the VIPD-Q. At a 3-month follow-up, we found that the VIPD-Q score declined in the post-DBS group. As oculomotor parameters were associated with axial symptoms and low frequency was reported to have a stronger effect on axial symptoms (Sidiropoulos et al., 2013; Xie et al., 2018), we compared groups with different stimulation parameters (voltage, pulse, frequency), which did not seem to influence the VIPD-Q score.

The role of NMS in PD progression has been discussed in many articles (Takeda et al., 2014; Hughes et al., 2018; Postuma et al., 2019; Cryan et al., 2020). However, few studies have focused on associations between NMS. We presumed that hyposmia would relate to visual dysfunction, as the olfactory and optic nerves are anatomically close. Strikingly, the hyposmia group in our study did not show a higher percentage of visual impairment than the control group. In contrast, the RBD-positive group showed a higher risk of visual impairment, consistent with a recently published study (Yang et al., 2016). Another vital finding in our investigation is that constipation was strongly correlated with visual impairment (percentage of visual impairment, 89 vs. 19%, *p* < 0.001). Thus, visual dysfunction in PD may originate peripherally.

Total VIPD-Q scores correlated with MoCA scores in our study, consistent with the well-established knowledge that visual dysfunction is associated with cognitive impairment. For example, thinner RNFL has predictive value as a biomarker of cognitive decline in PD (Murueta-Goyena et al., 2021). As VIPD-Q is negatively related to RNFL thickness, we consider that retinal neurodegeneration, neuronal loss, and anomalous α-synuclein deposits within the inner retinal layers may mediate this association. This hypothesis was supported by another study (Veys et al., 2019). Interestingly, the bias of thickness mainly occurred in the superior and inferior quadrants, as was observed in other Lewy body studies (Garcia-Martin et al., 2014; Murueta-Goyena et al., 2019, 2021).

Previous studies have reported that ocular microvascular patterns differ in patients with PD compared to healthy controls (Rascunà et al., 2020; Tsokolas et al., 2020; Robbins et al., 2021). We developed a software to extract the vessel from ocular fundus photography and analyze its morphology. The software was developed based on the U-Net model and uploaded to GitHub for free use. Our results showed that vessel density declined in the cognitively impaired group. This may be another explanation for the correlation between VIPD-Q scores and cognitive impairment. We also found that the PVS count in the higher VIPD-Q group exceeded that in the control group. Recent studies have indicated that PVS plays a disease-predicting role in PD, affecting cognitive status (Shibata et al., 2019; Rascunà et al., 2020; Shen et al., 2021) and motor prognosis (Chung et al., 2021). Our study demonstrated a relationship between vessel density and PVS count in PD, which, to our knowledge, has not been reported previously.

Our study was strengthened by a relatively large sample size and extensive sequencing data. This study provides powerful evidence for the positive effects of DBS on visual function. We propose a novel hypothesis that dysbiotic gut microbiota correlates with visual dysfunction in PD. Further investigation is needed to determine whether probiotics or antibiotics may help patients with PD avoid visual impairment. PVS was associated with vessel density in the ocular fundus. This work has clinical implications, as our data suggest that ophthalmic parameters are tightly correlated with cognitive status in patients with PD. Clinicians can potentially predict the risk of cognitive impairment in PD precisely and conveniently using a nomogram.

The hypothesis that visual impairment in PD originates from dysbiosis of microbiota should be investigated further through cell-based functional studies. In addition, the follow-up time was up to 3 months, and patients with PD receiving STN-DBS may reduce their levodopa dose after this time point. As levodopa was reported to rescue retinal morphology and visual function in a murine model of human albinism (Lee et al., 2019; Vagge et al., 2020), further investigation is needed to determine whether the reduction of levodopa after DBS aggravates visual impairment. Another limitation is that 50% of the PD participants were recruited by the neurosurgery department while waiting to undergo a DBS operation. This may create selection bias regarding indications for DBS, including disease duration over 3 years and sensitivity to the levodopa challenge test. Finally, the nomogram model would benefit from external validation in a separate PD population.

In conclusion, our study demonstrated that VIPD-Q can be applied to the Chinese population and is a useful tool for screening visual impairment among patients with PD. DBS showed a positive effect on PD patients' visual function, and visual impairment was linked with cognitive decline. Thinner RNFL and lower vessel density in the ocular fundus may explain this correlation. Our study indicated that visual impairment in patients with PD may originate from the dysbiotic gut microbiota. This conclusion strongly supports the presence of interactions between gut–eye and gut–brain axes.

## Data availability statement

The original contributions presented in the study are included in the article/Supplementary material, further inquiries can be directed to the corresponding author.

## Ethics statement

The studies involving human participants were reviewed and approved by the Ethics Committees at Qilu Hospital (protocol KYLL-202008-065) and the First Affiliated Hospital of Shandong First Medical University (protocol S569) approved this study. The patients/participants provided their written informed consent to participate in this study. Written informed consent was obtained from the individual(s) for the publication of any potentially identifiable images or data included in this article.

## Author contributions

CZ and HW performed the questionnaire including VIPD-Q, UPDRSIII, and MoCA. XW and Q-qW evaluated the status of movement disorders and made the statistics. All authors contributed to the article and approved the submitted version.

## Conflict of interest

The authors declare that the research was conducted in the absence of any commercial or financial relationships that could be construed as a potential conflict of interest.

## Publisher's note

All claims expressed in this article are solely those of the authors and do not necessarily represent those of their affiliated organizations, or those of the publisher, the editors and the reviewers. Any product that may be evaluated in this article, or claim that may be made by its manufacturer, is not guaranteed or endorsed by the publisher.

## Abbreviations

PD: Parkinson's Disease
VIPD-Q: Visual Impairment in Parkinson's Disease Questionnaire
DBS: Deep Brain Stimulation
RBD: rapid eye movement (REM) sleep behavior disorder
OCT: Optical Coherence Tomography
RNFL: Retinal Nerve Fiber Layer
VPA: vessel percentage area
UPDRS III: Unified Parkinson's Disease Rating Scale Section III
MoCA: Montreal Cognitive Assessment.
