# Supplementary figures and images for: Ophthalmologic problems correlates with cognitive impairment in patients with Parkinson's disease
Source: Front Neurosci. 2022 Oct 6;16:928980. doi: 10.3389/fnins.2022.928980 (PMC9583907; doi:10.3389/fnins.2022.928980)

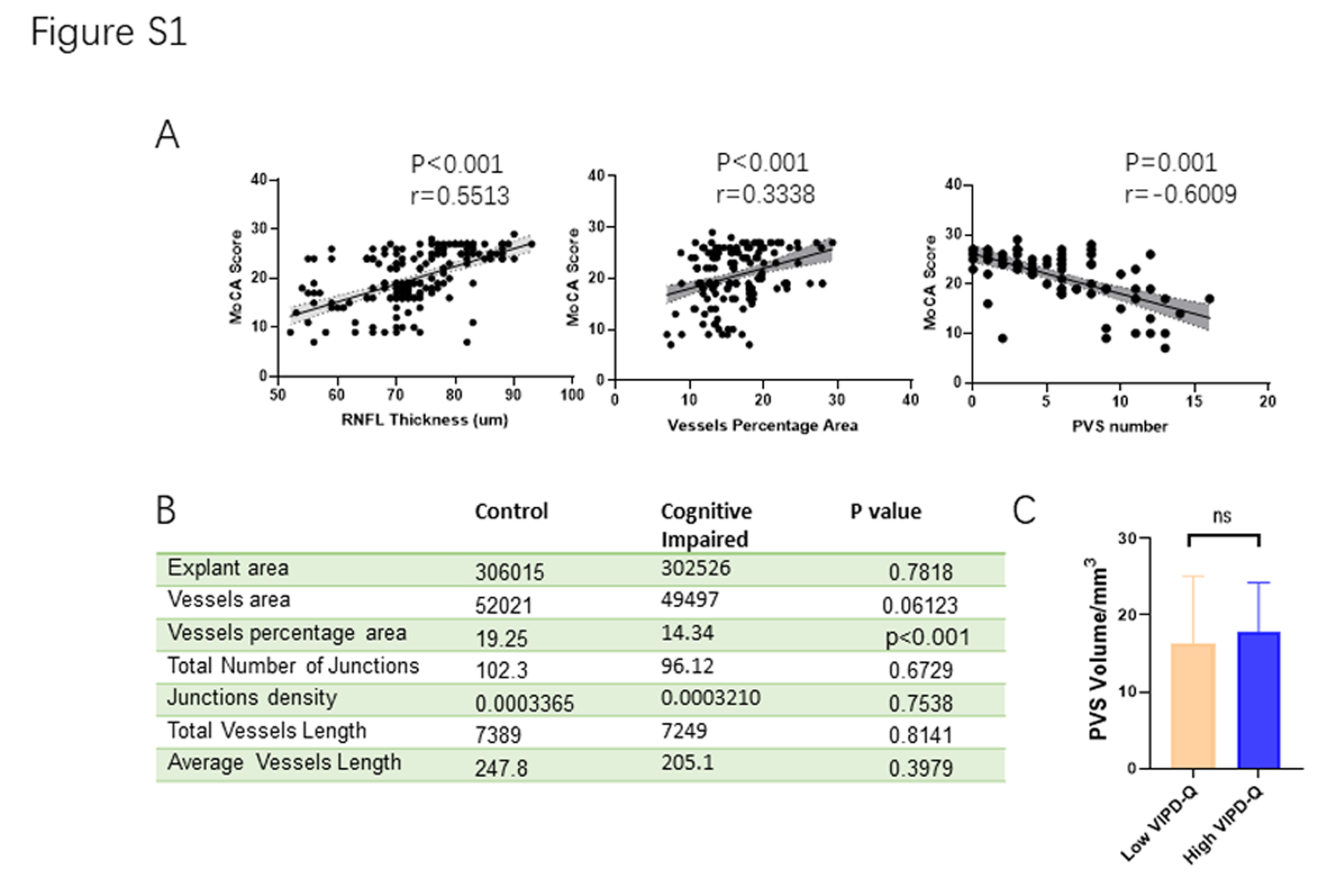

Supplement: Supplementary file 1 [file Image_1.TIF]
